# Will People Accept a Third Booster Dose of the COVID-19 Vaccine? A Cross-Sectional Study in China

**DOI:** 10.3389/fpubh.2022.914950

**Published:** 2022-07-12

**Authors:** Yufang Sun, Hang Dai, Ping Wang, Xiaodong Zhang, Dongliang Cui, Yongping Huang, Jimei Zhang, Tao Xiang

**Affiliations:** Emergency Department, The Third People's Hospital of Chengdu, The Affiliated Hospital of Southwest Jiaotong University, The Second Affiliated Hospital Chengdu Clinical College of Chongqing Medical University, Chengdu, China

**Keywords:** COVID-19, vaccine, acceptance, third booster dose, Chinese people

## Abstract

**Objective:**

The coronavirus disease 2019 (COVID-19) vaccines are considered to be an effective way to prevent the spread of the infection. Our previous study has shown that about 75% of healthcare workers (HCWs) in China were willing to receive the vaccine when it became available. Here, we examined the acceptance of a third booster dose among Chinese people and identified the influencing factors.

**Methods:**

A cross-sectional online survey was conducted and the snowball sampling method was utilized. An online questionnaire was provided to all the participants in the form of a quick response (QR) code. The questionnaire included general demographic information, views on vaccines, the General Health Questionnaire-12 (GHQ-12), and the Depression, Anxiety, and Stress Scale-21 (DASS-21). The univariate analysis was done between all the variables and our dependent variable. Then, we used the multivariate logistic regression model to examine the influencing factors of the third booster dose acceptance.

**Results:**

We collected 1,062 complete answers. Of these, 90.39% (*n* = 960) declared that they would accept the booster dose. Knowing more about the vaccine and recognizing the efficacy of vaccines were significantly associated with greater acceptance of the booster dose. People willing to take the booster dose had better psychological health. A belief that the booster dose could prevent severe infection caused by COVID-19 and enhance the effectiveness of the first two doses were the main contributing factors to vaccine acceptance. Vaccine hesitancy was mainly due to a low perceived risk of severe acute respiratory syndrome coronavirus 2 (SARS-CoV-2) infection and rapid mutation of SARS-CoV-2.

**Conclusion:**

This study revealed that Chinese people were very receptive to the third booster dose, which is an inspiring result. More positive attitudes regarding COVID-19 vaccination were supported by its efficacy and few side effects.

## Introduction

The ongoing pandemic of coronavirus disease 2019 (COVID-19) has caused extensive damage worldwide. Despite established supportive therapies and the development of new antiviral drugs, vaccination is considered an effective method to prevent infection of severe acute respiratory syndrome coronavirus 2 (SARS-CoV-2), particularly for at-risk populations. In China, the COVID-19 vaccines started to be used in priority groups on 15 December 2020. The Chinese government accelerated free vaccination for all the Chinese citizens starting in late March 2021 (1). As of 18 February 2022, more than three billion COVID-19 vaccine doses had been given nationwide and more than 1.2 people had completed the two-dose regimen (2).

However, typically, vaccine-induced immunity diminishes over time. Also, newly emerging SARS-CoV-2 variants can evade the immunity primed with vaccines developed against older variants (3). Hence, the need for a third dose of the COVID-19 vaccine has been discussed (4).

Increasing numbers of studies have suggested that a third booster dose could induce robust cellular and humoral immunity, thereby mitigating the fading of neutralizing antibodies after inoculation with two doses and reducing the risk of SARS-CoV-2 infection (5–9). A systematic review of 30 published studies on the efficacy and safety of the third dose of the COVID-19 vaccine has suggested that the reduction in the risk of infection ranged from 88 to 92% and conversion rates for immunoglobulin G ranged from 95 to 100% (10). Also, in cancer cases or immunocompromised patients, a significant increase in the antibody titer was noted (10). Studies have also shown that the third dose of the COVID-19 vaccine can reduce the risk of severe illness (5). Meanwhile, the safety of the third dose of the COVID-19 vaccine has been shown in several studies. A study about the safety and immunogenicity of the third dose in Chinese adults indicated that the third dose with either homologous or heterologous vaccine showed favorable safety profiles (3). A randomized controlled trial has shown that there were no serious side effects within 28 days after the third dose (9). Alasdair Munro et al. have assessed the safety of seven COVID-19 vaccines as a third dose and found that serious adverse events were uncommon and similar in active vaccine and control groups (11). Although all the studies mentioned above revealed the safety and immunogenicity of the third dose of a COVID-19 vaccine, whether people are willing to receive the third dose of the COVID-19 vaccine is still unknown. A total of 54.6% of Chinese people reported “probably yes” regarding their intent to accept the COVID-19 vaccine before it was developed (12). We previously reported that only one-third of healthcare workers (HCWs) in China were willing to take the COVID-19 vaccine when it became available (13). A total of 58.2% of medical students reported vaccine hesitancy months after the COVID-19 vaccine became available for use (14). Here, we examined the acceptance of the third booster dose among Chinese people and assessed the factors associated with its acceptance.

## Materials and Methods

### Study Participants

The study protocol was approved (2022-S-27) by the Ethics Committee of Third People's Hospital of Chengdu (Chengdu, China). Informed consent was obtained before study enrollment. This was a cross-sectional online survey using a social media platform (WeChat™)-based survey program “Questionnaire Star” between 1 December and 31 December 2021. The online questionnaire was provided to all the participants in the form of a quick response (QR) code *via* WeChat. The snowball sampling method was utilized; thus, agreeable participants could send the QR code to their respective WeChat friends in the same way. Participants answered the questions on the questionnaire by scanning the QR code. Participation was voluntary and the responses were anonymous. All the adults (>18 years of age), regardless of region, occupation, or status, were eligible to participate in our study.

### Questionnaire Design and Data Collection

Before initiating the study, we first consulted psychologists working at the Third People's Hospital of Chengdu and the psychologists recommended two commonly used scales, as described below, suitable for measuring psychological status. The questionnaire gathered information on: (i) general demographics (e.g., sex, age, education level, occupation, marital status, children, and income); (ii) whether or not to accept the third booster dose; and (iii) the reasons for being willing or not willing to be vaccinated. Moreover, this questionnaire included the General Health Questionnaire-12 (GHQ-12) and the Depression, Anxiety, and Stress Scale-21 (DASS-21) to investigate the psychological health of respondents.

Participants were asked if they would accept the third booster dose of the COVID-19 vaccine. They rated the item regarding their attitudes toward a booster dose on a four-point scale (1 = strongly agree, 2 = agree, 3 = disagree, and 4 = strongly disagree). Those answering 1 or 2 were identified as the vaccine-agree group. Those who answered 3 or 4 were identified as the vaccine-disagree group. Then, we listed common or possible causes of people's willingness or unwillingness to be vaccinated. People who agreed to take the vaccine could voluntarily select factors promoting their willingness to be vaccinated. Those who did not agree to take the vaccine could voluntarily choose the factors causing their resistance. The percentage of people who chose different factors was calculated.

With the help of a consultant psychologist, we chose the scores for the GHQ-12 and the DASS-21 to measure participants' mental health. The GHQ-12 is widely used to identify common psychiatric conditions (15, 16). The questionnaire consists of 12 items, where each is assessed with a four-point Likert scale ranging from “never” to “often” and is used with the 0–0–1–1 scoring method. The total score ranges between 0 and 12 points (poor mental health was defined as a total score ≥3) (17). The higher the score, the more significant the mental problem. The DASS-21 is a popular measure of mental health (18, 19). It consists of the subscales of depression, anxiety, and stress with 21 items (seven items for each subscale). Each seven-item subscale is rated on a four-point Likert scale ranging from 0 (“Did not apply to me at all”) to 3 (“Applied to me very much”). The higher the score, the more significant is the mental problem (20, 21).

Only complete questionnaires could be collected and incomplete data could not be submitted through the Questionnaire Star. The Questionnaire Star automatically collected data. We could convert all the data into text format and numeric form and export them to spreadsheets.

### Statistical Analysis

Descriptive analyses were conducted on all the study variables, which were reported as the mean, SD, number (*n*), and percentage. We used the univariate analysis with the *t*-test or the chi-squared test to compare the two groups (agree and disagree) to identify the factors associated with vaccine hesitancy. Then, the multivariate logistic regression model was employed to examine and identify the factors associated with the acceptance of the COVID-19 vaccine. Statistical analyses were performed using SPSS version 23.0 (IBM Incorporation, Armonk, New York, USA). *p* ≤ 0.05 was considered statistically significant.

## Results

### Acceptance of the Third Booster Dose

We collected 1,062 responses. Of these, 960 responders (90.39%) declared that they would accept a booster dose. Of those who were willing to accept the third booster dose, 884 responders (83.2%) were strongly willing and 76 responders (7.1%) were willing but waiting to review more data. Of the 1,062 participants, only 102 (9.6%) participants were not willing to accept the third booster dose. Of them, 71 (6.7%) participants did not plan to receive the COVID-19 vaccine and 31 (2.9%) participants were strongly against taking the booster dose. We found that 95.6% (1,016) of respondents had completed the two-dose regimen and 97.3% (935) of respondents who had completed the two doses were willing to receive the third dose (Table 1).

**Table 1:** Demographics of study population and the univariate analysis between the agree and disagree groups (*n* = 1,062).

**Variable**	***n* = 1,062 (percentage)**	**Agree group (*n* = 960)** **(percentage)**	**Disagree group (*n* = 102)** **(Percentage)**	**χ^2^*/t***	***p*-Value**
**Sex**					
Male	406 (38.2)	371 (38.6)	35 (34.3)	0.733	0.392
Female	656 (61.8)	589 (61.4)	67 (65.7)		
**Age (years)**					
<18	3 (0.3)	2 (0.2)	1 (1.0)	3.825	0.407
18–30	377 (35.5)	346 (36.1)	31 (30.4)		
31–40	305 (28.7)	272 (28.4)	33 (32.4)		
41–50	196 (18.5)	177 (18.4)	19 (18.6)		
>50	181 (17.0)	163 (16.9)	18 (17.6)		
**Healthcare workers**					
Yes	497 (46.7)	457 (47.6)	40 (39.2)	2.606	0.106
No	565 (53.3)	503 (52.4)	62 (60.8)		
**Education**					
Junior high and below	93 (8.7)	86 (8.9)	7 (6.9)	4.785	0.187
Senior school	97 (9.1)	90 (9.3)	7 (6.9)		
Bachelor	724 (68.1)	657 (68.5)	67 (65.7)		
Postgraduate	148 (13.9)	127 (13.3)	21 (20.5)		
**Marital status**					
Married	725 (68.2)	652 (67.9)	73 (71.6)	0.568	0.451
Single	337 (31.8)	308 (32.1)	29 (28.4)		
**Children**					
Yes	682 (64.2)	621 (64.6)	61 (59.8)	0.957	0.328
No	380 (35.8)	339 (35.4)	41 (40.2)		
**Living with elderly individuals**					
Yes	538 (50.6)	493 (51.3)	45 (44.1)	1.932	0.165
No	524 (49.4)	467 (48.6)	57 (55.9)		
**Influenza vaccination in 2020**					
Yes	525 (49.4)	482 (50.2)	43 (42.2)	2.391	0.122
No	537 (50.6)	478 (4.8)	59 (57.8)		
**Worried about experiencing COVID-19**					
Yes	666 (62.7)	613 (63.8)	53 (52.0)	5.577	0.018*
No	396 (37.3)	347 (36.2)	49 (48.0)		
**Understanding how the vaccine works**					
Yes	1,017 (4.3)	924 (96.2)	93 (91.2)	5.849	0.016*
No	45 (95.7)	36 (3.8)	9 (8.8)		
**Completion of COVID-19 vaccination with a two-dose regimen**					
Yes	1,016 (95.6)	935 (97.3)	81 (79.4)	71.965	<0.001**
No	46 (4.4)	25 (2.7)	21 (20.6)		
**The vaccine is effective**					
Yes	925 (87.1)	867 (90.3)	58 (56.9)	91.816	<0.001**
No	137 (12.9)	93 (9.7)	44 (43.1)		
**COVID contact or living in a high-risk area**					
Yes	86 (8.1)	81 (8.4)	5 (4.9)	1.549	0.213
No	976 (91.9)	879 (91.6)	97 (95.1)		
**Income (RMB)**					
<5,000	412 (38.8)	381 (39.6)	31 (30.4)	4.034	0.134
5,000–10,000	465 (43.7)	417 (43.5)	48 (47.1)		
>10,000	185 (17.5)	162 (16.9)	23 (22.5)		
**Effect of COVID-19**					
Not at al	40 (3.7)	38 (3.9)	2 (2.0)	6.842	0.086
Mild	67 (6.3)	55 (5.7)	12 (11.8)		
Moderate	581 (54.7)	528 (55.1)	53 (51.9)		
Severe	374 (35.3)	339 (35.3)	35 (34.3)		
**GHQ-12 score**					
≥3	105 (9.9)	88 (9.2)	17 (16.7)	5.821	0.016*
<3	957 (90.1)	872 (90.8)	85 (83.3)		
**DASS-21**					
Depression (mean ± SD)	1,062	3.61 ± 3.18	5.75 ± 4.10	9.378	0.002*
Anxiety (mean ± SD)	1,062	2.71 ± 3.25	3.93 ± 4.33	17.03	<0.001**
Stress (mean ± SD)	1,062	3.73 ± 3.77	4.96 ± 4.21	2.475	0.116

^*^p < 0.05.

^**^p < 0.001.

### Variables Associated With Acceptance of the Third Booster Dose

The univariate analysis was used to decide significant differences between the two groups (the agree and disagree groups; Table 1). There were significant differences among the respondents in worrying about experiencing COVID-19, understanding how the vaccine works, completing a two-dose regimen, thinking that the vaccine works, and having good mental health. Results of the univariate analysis are shown in Table 1. Individuals willing to receive the booster dose were more worried about infection (χ^2^ = 5.577, *p* = 0.018), had a better understanding of how the vaccine works (χ^2^ = 5.849, *p* = 0.016), had nearly completed a two-dose regimen, were more likely to think that the vaccine works (χ^2^ = 91.816, *p* = 0.000), and had better mental health based on the GHQ-12 (χ^2^ = 14.805, *p* = 0.002; depression: *F* = 9.378, *p* = 0.002; anxiety: *F* = 17.03, *p* = 0.000). However, the two groups did not differ significantly in terms of sex, age, occupation, educational level, marital status, contact with a person with COVID-19, living in a high-risk area, or income.

To determine the factors associated with the willingness to be vaccinated, we used the multivariate logistic regression models. Results are given in Table 2. Five factors were significantly associated with the acceptance of the third booster dose: (a) worried about experiencing COVID-19; (b) understanding how the vaccine works; (c) completion of COVID-19 vaccination with a two-dose regimen; (d) the vaccine is effective; and (e) the GHQ-12 score. Those with a high perceived risk of being infected had twice the odds of vaccine acceptance compared with those with no perceived risk of being infected [odds ratio (OR) = 2.159; 95% CI = 1.331–3.501; *p* = 0.002]. Additionally, those who knew more about vaccine properties were almost three times more likely to accept the third booster dose (OR: 2.879; 95% CI: 1.143–7.148; *p* = 0.025). People who had completed the two-dose regimen were more likely to accept the third dose compared with those who had not completed the two-dose regimen (OR: 9.708; 95% CI: 4.474–21.069; *p* = 0.000). People who believed that the vaccine was effective had 7.949 times greater odds of accepting the third dose compared with those who believed that the vaccine was not effective (OR: 7.949; 95% CI: 4.769–13.251; *p* = 0.000). People with the lower GHQ-12 score were more likely to accept the third dose (OR: 2.903; 95% CI: 1.476–5.708; *p* = 0.002) compared to those with the higher GHQ-12.

**Table 2:** The multivariate logistic regression analyses showing the factors associated with acceptance of a booster dose (*n* = 1,062).

**Variable**	**OR**	**95%CI**	***p*-Value**
**Sex**			
Male (reference)	1		
Female	0.673	0.401–1.130	0.134
**Age (years)**			
<18 (reference)	1		
18–30	0.372	0.021–6.517	0.498
31–40	1.409	0.539–3.687	0.484
41–50	1.201	0.556–2.596	0.641
>50	0.862	0.388–1.915	0.716
**Healthcare workers**			
Yes (reference)	1		
No	0.694	0.409–1.180	0.177
**Education**			
Junior high and below (reference)	1		
Senior school	2.225	0.687–7.198	0.182
Bachelor	2.220	0.753–6.550	0.148
Postgraduate	1.611	0.868–2.991	0.131
**Marital status**			
Married (reference)	1		
Single	2.099	0.940–4.690	0.071
**Children**			
No (reference)	1		
Yes	1.913	0.864–2.488	0.122
**Living with elderly individuals**			
No (reference)	1		
Yes	1.466	0.824–2.448	0.156
**Influenza vaccination in 2020**			
No (reference)	1		
Yes	1.215	0.756–1.950	0.421
**Worried about experiencing COVID-19**			
No (reference)	1		
Yes	2.159	1.331–3.501	0.002*
**Understanding of the vaccine**			
No (reference)	1		
Yes	2.879	1.143–7.248	0.025*
**Completion of COVID-19 vaccination with a two-dose regimen**			
No (reference)	1		
Yes	9.708	4.474–21.069	<0.001**
**The vaccine is effective**			
No (reference)	1		
Yes	7.949	4.769–13.251	<0.001**
**COVID contact or living in a high risk area**			
No (reference)	1		
Yes	1.659	0.607–4.533	0.323
**Income (RMB)**			
<5,000 (reference)	1		
5,000–10,000	1.662	0.747–3.694	0.213
>10,000	1.007	0.524–1.935	0.984
**GHQ score**			
≥3 (reference)	1		
<3	2.903	1.476–5.708	0.002*

^*^p < 0.05.

^**^ p < 0.001. OR, odds ratio; CI, confidence interval.

### Reasons for Willingness or Unwillingness to Be Vaccinated

**Figure 1:**
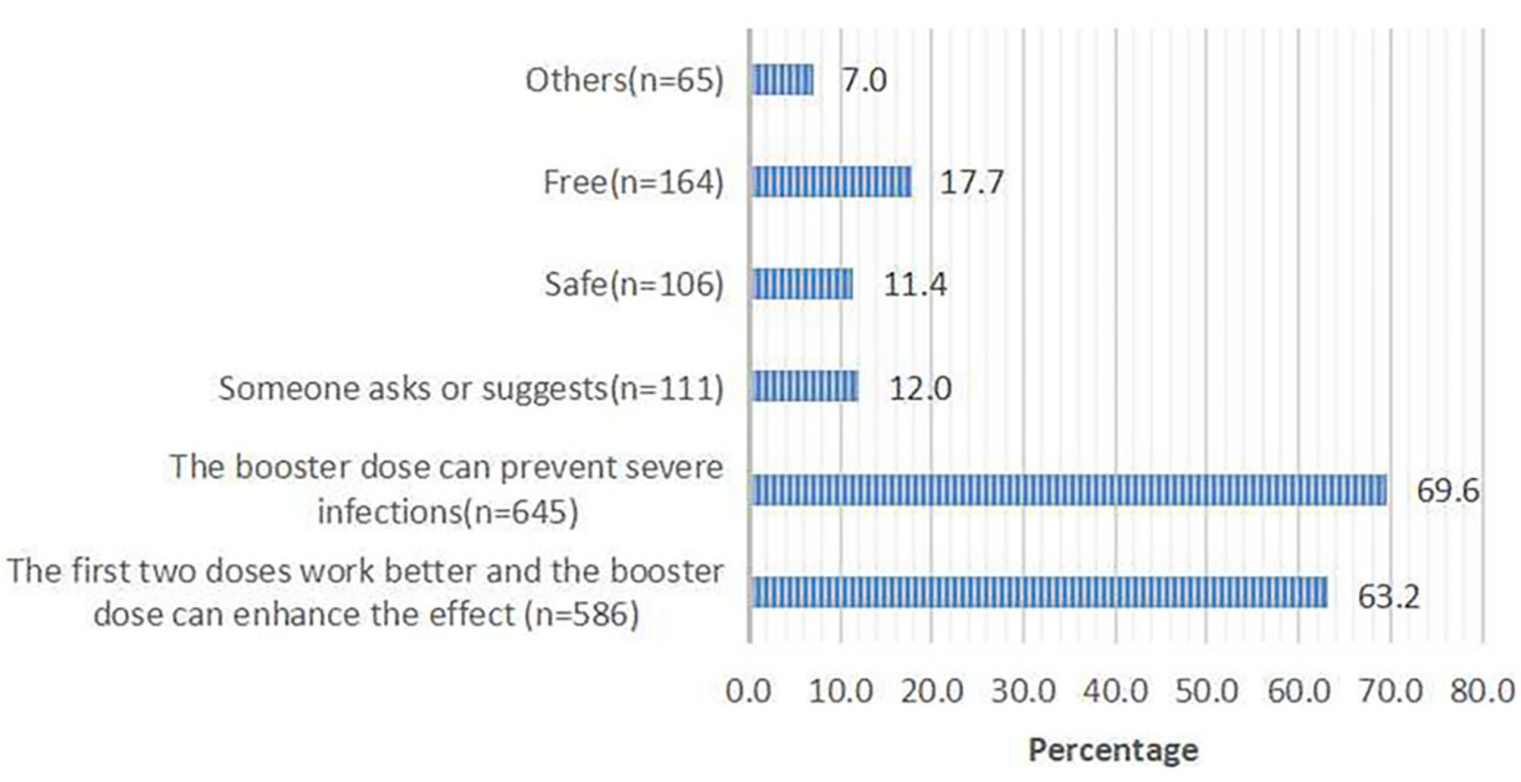
The main contributing factors for taking the third booster dose; responses from 960 participants who said they would accept the booster dose.

**Figure 2:**
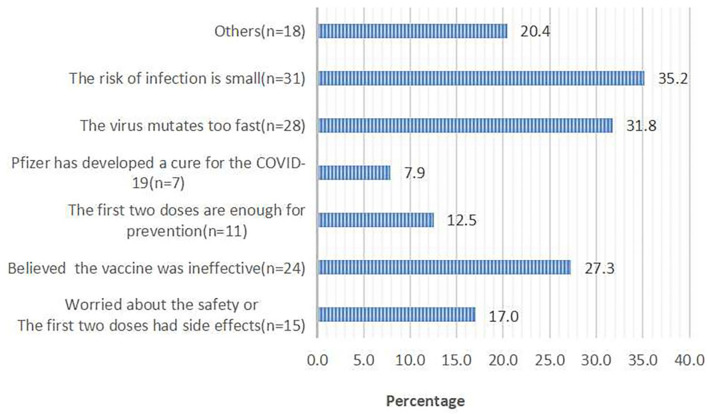
Reasons for hesitancy in taking the third booster dose; responses from 102 participants who said they would refuse the booster dose.

Of 960 respondents who were willing to receive the third dose, 927 (96.6%) respondents chose the reasons for accepting the third dose. Of 102 respondents who were not willing to receive the third dose, 88 (86.3%) respondents chose the reasons for rejecting the third dose. The top two reasons for accepting the third dose were that the booster dose can prevent severe infections (69.6%) and enhance the effect of the first two doses (63.2%; Figure 1). The top two reasons for rejecting the third dose were a perceived low risk of infection (35.2%) and rapid mutation of SARS-CoV-2 (31.8%; Figure 2).

## Discussion

Despite newly approved antiviral drugs, the role of vaccination remains crucial. However, the efficacy of vaccination diminishes over time. Cohn and their colleagues have demonstrated that the efficacy of the COVID-19 vaccine against infection declined from 87 to 48% from February to October 2021 (22). Protection levels against SARS-CoV-2 (including the Delta variant) infection or symptomatic infection decline over time (23–25). Therefore, policymakers have begun to consider using the third booster dose to improve protection.

Most surveys on COVID-19 have focused on the safety and immunogenicity of the third dose. Only a few studies have assessed the acceptance of the third booster dose. Suman and their colleagues have found that nearly two-thirds of respondents were concerned that vaccination may be ineffective against new strains of SARS-CoV-2 and that booster doses may be required. However, acceptance by vaccine-hesitant respondents of a hypothetical booster dose was only 14.3% (26). Another study has indicated that 84.5% of medical students were willing to receive the third dose of the COVID-19 vaccine (4). Those studies were aimed at HCWs and were conducted from August to October 2021. However, after those studies, an increasing number of studies have demonstrated the efficacy and safety of the third booster dose. Hence, what are the attitudes of the general population toward the booster dose? What are the influencing factors? Our study provided some answers to those questions.

In our study, 90.39% of participants declared that they would accept the booster dose. This acceptance rate was higher than that of the first two doses in our previous study (76.63%) (13) among HCWs in the USA (36%) (27), among the general adult population in Kuwait (53.1%) (28), among citizens of the Democratic Republic of Congo (55.9%) (29), and among the general population in some other low- and middle-income countries in Asia and Africa (55%−80%) (30). Studies have found major barriers concerning the safety and efficacy of the COVID-19 vaccine and the rapid mutation of SARS-CoV-2 (13, 31). However, the first two factors became the motivator for the third booster dose in the present study. Having a low perceived risk of COVID-19 was an important factor for vaccine hesitancy, an issue not addressed in previous studies except in one review, in which the author has drawn a similar conclusion by analyzing some phenomena among Muslims (32). This perception may occur because people felt that they had already benefited from the first two doses and surveillance and control measures had been taken by the government. Additionally, worrying about the rapid mutation of SARS-CoV-2 was one of the primary factors making people hesitant about taking the booster dose. We included anti-COVID-19 drugs as a factor in the options and 7.9% of participants chose it as a factor, most of whom concurrently chose the factor “worried about the efficacy and safety of vaccines.” This finding suggested that some people prefer to believe that drugs, rather than vaccination, can be used to treat COVID-19, which may be related to the safety and side effects of vaccines and the uncertainty of the efficacy of vaccines.

Our study indicated that people with a high perceived risk of being infected had twice the odds of vaccine acceptance compared with those without a perceived risk of being infected. This concern is not surprising given that vaccines remain the main protection method against COVID-19. This result is similar to the findings noted by Harapan and collaborators and Rajamoorthy and coworkers (33, 34). Another study has also found that a high perceived risk of COVID-19 was associated with the acceptance of the COVID-19 vaccine among HCWs in China (35). We found that ~40% of respondents did not worry about experiencing COVID-19. This observation may be, at least in part, due to the effective control of the COVID-19 epidemic in China. In China, everyone was required to wear a face-covering mask outdoors during the COVID-19 epidemic. Especially during the COVID-19 epidemic outbreak, people were forbidden to gather in a public places, such as karaoke, bars, or movie venues, workers were encouraged to hold online meetings, and frontline HCWs were asked to take PCR tests twice a week. Hence, effective protective measures are also extremely important besides vaccination.

Our study indicated that learning more about the COVID-19 vaccine might have contributed to people being more willing to take the vaccine compared with those who had known less about the vaccine. This finding is consistent with data from our previous study, suggesting that greater education efforts toward vaccination against COVID-19 should be considered to increase public understanding of vaccines. People who believed that the COVID-19 vaccine was effective had 7.949 times greater odds of accepting the third dose compared with those who believed that the COVID-19 vaccine was not effective. Harapan and colleagues have reported similar findings (33). We found that approximately 90% of respondents thought that the COVID-19 vaccine was efficacious, showing that the efficacy of the COVID-19 vaccine was accepted widely. Importantly, people who had completed the two-dose regimen were more likely to accept the third dose compared with those who had not completed the two-dose regimen, as also suggested by Sugawara et al. (4). Those data suggest that: (i) the first two doses are widely approved and (ii) if there is sufficient evidence to demonstrate the efficacy and safety of the COVID-19 vaccine, then people will be more willing to be vaccinated. Wheelock and their coworkers have studied the psychological factors underlying adult behavior toward the influenza vaccine. They have revealed that a better understanding of the psychological aspects of vaccination across contexts and vaccines is a priority (36). However, no studies have linked psychological factors to the acceptance of COVID-19 vaccination except in our previous study. In the present study, we included the questionnaire internationally recognized scales to assess mental health. We found that people with the lower GHQ-12 score were more likely to accept the third dose. This observation suggests that people who were willing to take the booster dose had better psychological health. However, the low mental health status may be related to many factors (e.g., illness, living conditions, and work pressure), which may affect vaccine acceptance.

Our study had two major strengths. First, it was the first study to evaluate the acceptance of the third booster dose among the general population in China. Second, we evaluated mental health to investigate if it influenced vaccine hesitancy.

Our study had two main limitations. First, we employed an electronic questionnaire to collect data (instead of a face-to-face interview), which resulted in uncontrolled conditions during questionnaire completion. Second, as we used a snowball sampling method, some individuals in this sample have still not been found and some individuals are probably omitted by a provider, leading to a biased sample. Third, the sample size was small, limiting the generalizability of our findings.

## Conclusion

In China, about 1.2 billion people have completed the two-dose regimen. The safety and efficacy of the COVID-19 vaccines have been recognized. People's acceptance of the booster dose has also been improved. Our study findings make us optimistic about the COVID-19 vaccination. We believe that the COVID-19 vaccination campaign will progress smoothly and will eventually provide herd immunity against COVID-19.

## Data Availability Statement

The original contributions presented in the study are included in the article/supplementary material, further inquiries can be directed to the corresponding author.

## Ethics Statement

The studies involving human participants were reviewed and approved by the Ethics Committee of Third People's Hospital of Chengdu.

## Author Contributions

YS, TX, PW, and XZ conceived and designed the questionnaire. JZ, DC, and YH recruited participants. YS, TX, and HD analyzed the data. YS wrote and revised the manuscript. All authors have approved the final version of the manuscript and agreed with submission to your esteemed journal.

## Conflict of Interest

The authors declare that the research was conducted in the absence of any commercial or financial relationships that could be construed as a potential conflict of interest.

## Publisher's Note

All claims expressed in this article are solely those of the authors and do not necessarily represent those of their affiliated organizations, or those of the publisher, the editors and the reviewers. Any product that may be evaluated in this article, or claim that may be made by its manufacturer, is not guaranteed or endorsed by the publisher.
